# What are the health consequences associated with differences in medical malpractice liability laws? An instrumental variable analysis of surgery effects on health outcomes for proximal humeral facture across states with different liability rules

**DOI:** 10.1186/s12913-022-07839-0

**Published:** 2022-05-03

**Authors:** Brian Chen, Sarah Floyd, Dakshu Jindal, Cole Chapman, John Brooks

**Affiliations:** 1 Department of Health Services Policy and Management, University of South Carolina, 915 Greene Street Suite 354, Columbia, SC 29208 USA; 2 College of Behavioral, Social and Health Sciences, Clemson University, 116 Edwards Hall, Clemson, SC 29634 USA; 3 Department of Pharmacy Practice and Science, University of Iowa, 345 CPB, 180 South Grand Ave, Iowa City, IA 52242 USA; 4 Center for Effectiveness Research in Orthopaedics (CERortho), University of South Carolina, 915 Greene Street Suite 302, Columbia, SC 29208 USA

**Keywords:** Medical malpractice, Health law, Health outcomes, Orthopaedics, Proximal humeral fractures

## Abstract

**Background:**

States enacted tort reforms to lower medical malpractice liability, which are associated with higher surgery rates among Medicare patients with shoulder conditions. Surgery in this group often entails tradeoffs between improved health and increased risk of morbidity and mortality. We assessed whether differences in surgery rates across states with different liability rules are associated with surgical outcomes among Medicare patients with proximal humeral fracture.

**Methods:**

We obtained data for 67,966 Medicare beneficiaries with a diagnosis of proximal humeral fracture in 2011. Outcome measures included adverse events, mortality, and treatment success rates, defined as surviving the treatment period with < $300 in shoulder-related expenditures. We used existing state-level tort reform rules as instruments for surgical treatment and separately as predictors to answer our research question, both for the full cohort and for stratified subgroups based on age and general health status measured by Charlson Comorbidity Index and Function-Related Indicators.

**Results:**

We found a 0.32 percentage-point increase (*p* < 0.05) in treatment success and a 0.21 percentage-point increase (*p* < 0.01) in mortality for every 1 percentage-point increase in surgery rates among patients in states with lower liability risk. In subgroup analyses, mortality increased among more vulnerable patients, by 0.29 percentage-point (*p* < 0.01) for patients with Charlson Comorbidity Index >  = 2 and by 0.45 percentage-point (*p* < 0.01) among those patients with Function-Related Indicator scores >  = 2. On the other hand, treatment success increased in patients with lower Function-Related Index scores (< 2) by 0.54 percentage-point (*p* < 0.001). However, younger Medicare patients (< 80 years) experienced an increase in both mortality (0.28 percentage-point, *p* < 0.01) and treatment success (0.89 percentage-point, *p* < 0.01). The reduced-form estimates are consistent with our instrumental variable results.

**Conclusions:**

A tradeoff exists between increased mortality risk and increased treatment success across states with different malpractice risk levels. These results varied across patient subgroups, with more vulnerable patients generally bearing the brunt of the increased mortality and less vulnerable patients enjoying increased success rates. These findings highlight the important risk-reward scenario associated with different liability environments, especially among patients with different health status.

**Supplementary Information:**

The online version contains supplementary material available at 10.1186/s12913-022-07839-0.

## Introduction

Policy makers, legislators, and legal scholars often justify the tort liability system, of which medical malpractice liability is a part, on two grounds [1–5]. First, by connecting harm and liability, it compensates victims of iatrogenic harm. Second, the threat of costly liability awards could encourage medical providers to follow safe medical practices, yielding better health outcomes. Medical malpractice liability has also been faulted for distorting the practice of medicine by encouraging doctors to order more tests and procedures than medically necessary, [6] or shy away from complex patients[7] in order to manage medico-legal risk [8, 9]. However, the perception of rampant frivolous lawsuits led to waves of state tort reform laws beginning in the 1990s, with the purpose of limiting physician liability or otherwise increasing the difficulty of successful suits against medical providers [10–13]. As a result of these tort reform laws, healthcare providers across states practice under different tort law environments. In a previous paper, we demonstrated that this variation in the tort law environment was associated with differences in surgery rates for shoulder conditions among Medicare beneficiaries. States with laws leading to lower physician malpractice liability risk had higher surgery rates. The goal of this paper was to assess how the observed variation in surgery rates associated with malpractice liability laws affects patient health outcomes.

On one hand, it is possible that higher surgery rates lead to improved outcomes among patients by increasing access to needed care. Conversely, higher surgery rates associated with lower liability environments may extend surgery to riskier patients with higher risks of surgery-related adverse events and mortality. We used instrumental variable methods to exploit observed variation in surgery rates to directly assess the outcome implications for the subset of patients whose surgery decisions are associated with the medical malpractice law environment across states.

Existing empirical literature on medical malpractice, although vast, focused primarily on the relationship between the medical liability system and healthcare utilization or costs [14–23]. These studies rarely examined whether the resulting differences in utilization are associated with patient health outcomes. A recent meta-analysis identified 3,862 studies in the field, but only a handful studied relationships between tort law and healthcare outcomes [24]. This is an important gap in the literature. Existing studies suggest that physicians are more likely to depart from clinical standards of care in a low malpractice liability environment, with potentially significant implications for patient health and safety [25]. The limited empirical literature, however, generally does not have sufficient evidence to support this hypothesis [7, 26–28].

Our study builds upon related prior studies in several ways. First, we contribute to the comparatively limited literature on the on health outcome differences among patients across states with different medical liability rules. Second, to our knowledge, no study has evaluated such health outcome differences in orthopaedics specifically. This is an often overlooked medical specialty in the malpractice literature, but an important specialty that affects 1 in 2 Americans over the age of 18, and costs the U.S. healthcare system $176 annually in direct expenditures alone [29–31]. Moreover, orthopaedics is a field with remarkably little Level I evidence to guide treatment choices [32–47], signifying that any tort reform law-associated differences in practice patterns may have an unpredictable impact on patient safety and health outcomes.

Moreover, the few studies assessing the effect of tort reform laws on outcomes generally used difference-in-differences designs. These are strong empirical approaches, which exploit tort law changes in certain states. However, these approaches remain at risk of bias if tort law changes are spurred by conditions in each state related to patient outcomes. To complement results from these studies, we used surgery rate variation associated with entrenched cross-sectional variation in malpractice liability laws across states in an instrumental variable estimation [48] to assess the relationship between tort law-associated surgery choice and health outcomes. By “entrenched,” we mean that differences in surgical rates across states with different medical liability rules may theoretically have been caused by changes to these rules almost 20 years ago. That is, new generations of surgeons with more aggressive treatment styles may have been attracted to lower liability states, or new surgeons may have begun practicing more aggressively in reaction to reduced liability.

We also evaluated both beneficial and detrimental patient outcomes to understand the tradeoffs and heterogeneous impacts associated with these laws [49]. To our knowledge, none of the outcome studies assessed these relationships in patient subgroups. Subgroup analysis may yield important insights, as surgery may have heterogeneous effects on outcomes based on the patient’s baseline characteristics [50].

We specified the instrumental variables as a series of indicators representing different malpractice liability laws across states. [48] Based on prior research showing their effects on surgery rates for Medicare patients with proximal humeral fractures (PHF) [51], we chose four tort rules that reduced malpractice liability – joint and several liability reform (JSLR), caps on punitive damages, punitive evidence rule, and contributory negligence. Instrumental variable estimators provide estimates that are representative of PHF patients on the extensive margin – i.e., PHF patients whose surgery choice is sensitive to the tort reform rules in a state [49, 52–57]. We conducted the instrumental variable analysis on the entire sample, as well as different patient groups based on age, Charlson Comorbidity Index (CCI), and Function-Related Indicators (FRI) scores. We also estimated the corresponding reduced form equations by regressing our health outcomes of interest on the total number of liability-reducing tort rules.

## Methods

### Data and sample

We used data on 67,966 Medicare beneficiaries in the United States with a diagnosis of PHF in 2011 from the Research Data Assistance Center (ResDAC) previously analyzed elsewhere [51]. Radiography-confirmed patients with PHF were identified using diagnosis codes from Medicare Part B carrier, outpatient, and Medpar Part A inpatient claims. The full set of diagnosis codes used to identify PHF patients is included in Supplementary Appendix 1. To be included in the final analytical dataset, patients were required to have an incident diagnosis of PHF in 2011 and to survive a 60-day treatment period following the index date of PHF. Other inclusion and exclusion criteria are described in our prior work [51, 58–60] and in Appendix Table 1 as well. SAS version 9.4 (Cary, NC, USA) was used to build the analytical dataset.

**Table 1:** States by Combinations of Tort Rules

Contributory negligence	JSL	Cap on punitive damages	Punitive evidence rule	Mean surgery rates	N	States
0	0	0	0	0.088	1,999	MA
1	0	0	0	0.087	265	RI
0	1	0	0	0.15	2,114	CT VT WV WY
1	1	0	0	0.11	4,723	NM NY
1	0	1	0	0.16	5,681	IL VA
0	1	1	0	0.15	1,010	NE NH
1	1	1	0	0.19	4,879	LA MI WA
0	0	0	1	0.16	757	DE ME
1	0	0	1	0.14	1,562	DC MD
0	1	0	1	0.18	5,322	IA MN SC SD TN UT
1	1	0	1	0.16	6,595	AZ CA
0	0	1	1	0.16	1,939	IN
1	0	1	1	0.16	3,993	AL NC
0	1	1	1	0.17	19,174	AR CO GA ID KS MT ND NJ NV OH OK OR TX WI
1	1	1	1	0.18	8,004	FL MO MS

We disaggregated the full cohort of PHF patients into subgroups to assess the differential impact of varying state tort reform laws on health outcomes in different patient populations. These subgroups consisted of patients under 80 years of age versus those over 80; patients with CCI less than 2 or 2 and higher; and patients with FRI frailty score of less than 2 or 2 or higher.

### Treatment measure

Our treatment variable is an indicator denoting whether a patient underwent 1 of 4 surgical procedures (reverse shoulder arthroplasty, hemiarthroplasty, open reduction internal fixation, or closed reduction internal fixation) during the 60 days following the incident PHF diagnosis. These surgical procedures were identified using the Current Procedural Terminology codes in the Part B carrier, outpatient, and Medpar inpatient claims files. A complete list of these codes is provided in Supplementary Appendix Table 2.

### Tort reform law variables (Instruments)

As will be described further in the Analytical Approach section, we used an instrumental variable estimator to estimate the local average treatment effect of surgery on health outcomes. To be a valid instrument, our tort variables must first affect the likelihood of surgery. Here, we describe four tort laws that were shown previously to be associated with surgery rates [51], because a reduction in liability risk may encourage surgeons to recommend surgery for slightly riskier patients. JSLR is set to 1 if state law was reformed to require plaintiffs to sue only the healthcare providers most at fault rather than the medical team collectively. JSLR reduces malpractice liability risk for individual surgeons because patients may not know which provider to sue in today’s complex medical environment. “Contributory negligence” is set to 1 if a state bars a patient from suing a provider if the patient is at least minimally at fault for their own harm. “Cap on punitive damages” reduces surgeon liability risk by limiting on how much patients can receive for a provider’s gross negligence. Finally, “punitive evidence rule” also lowers surgeon liability risk because the evidentiary bar to obtain punitive damages against grossly negligent providers is set very high.

**Fig. 1:**
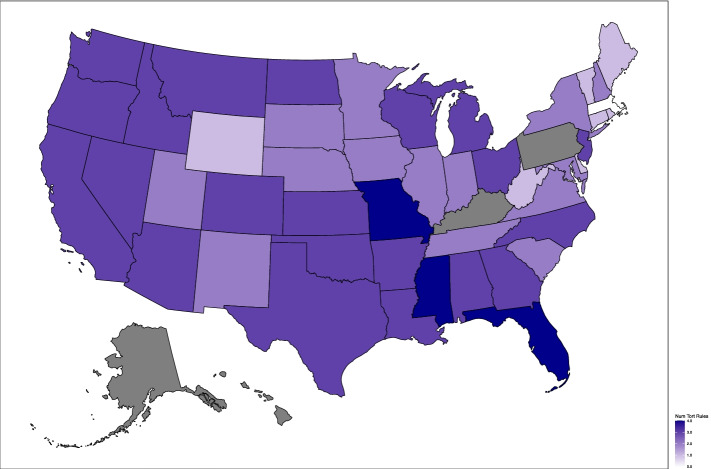
Number of State Tort Rules (2011)

We obtained data on state tort law liability regimes from the Database of State Tort Law Reforms, 1980–2018 (6^th^ Edition) from the University of Texas School of Law [61]. The status of the tort reform rules was determined based on each patient’s state of residence in 2011. We report the states with 0, 1, 2, 3 or all 4 of these tort laws in place in Table 1 and graphically in Fig. 1 to illustrate the geographic distribution of the states with various numbers of malpractice liability-lowering tort laws.

### Outcome measures

We defined the outcome period to run from day 61 through day 365 after the index PHF date. Because higher surgery rates can provide both benefits and detriments to PHF patients, we developed alterative outcome measures to capture both possibilities. With regard to detriments, our outcomes included any adverse events or death that occurred during the outcome period. An adverse event variable was set equal to 1 if a patient had at least one claim in the outcome period with diagnosis of a surgical or medical adverse event for PHF, 0 otherwise. Adverse events included infection, nerve injury, prosthetic complication, hematoma, avascular necrosis, adhesive capsulitis, shoulder instability or dislocation, pneumonia, cardiac dysrhythmias, congestive heart failure, and deep vein thrombosis or pulmonary embolism [59]. The dates of death were taken from the 2011 and 2012 Medicare Beneficiary summary files, and the variable mortality was set to 1 if the patient died during the outcome period, and set to 0 otherwise.

Finally, an “event-based [62] or “process of recovery” [63] measure of “treatment *success*” was derived from the Medicare claims [52, 62–71]. A “treatment success” variable was set equal to 1 if the patient survived the outcome period and had less than $300 in shoulder-related expenditures in the same period. Our clinical co-investigators suggested that even successfully treated PHF patients will use as many as four evaluation and management follow-up visits, with each visit costing an average of $75 in 2011. The $300 threshold was meant to capture patients whose shoulder condition required medical attention beyond usual follow-up, suggesting persistent shoulder-related issues.

### Covariates

We included relevant demographic and clinical information available in the Medicare database as covariates. Demographic characteristics included age, sex, race, dual eligibility for Medicare and Medicaid, Charlson Comorbidity Index (CCI) [72, 73], and Function-Related Indicator (FRI) scores [74]. Clinical variables included shoulder diagnoses of osteoarthritis, rheumatoid arthritis, rotator cuff arthropathy, and avascular necrosis, in addition to Medicare spending in the previous year. The detailed definitions and construction of all demographic and clinical covariates in our regressions is listed in Supplementary Appendix Table 2 and have been published elsewhere [60].

### Analytical approach

We used instrumental variable regressions because our goal was to assess outcome changes (treatment success, complications, mortality) resulting from physician behavior (surgery rates) that was sensitive to policy changes (tort reform laws). We believe that our tort reform rules satisfy both requirements for being good instruments. First, tort liability reform laws theoretically affect treatment choices because surgeons who face reduced malpractice risks are likely to increase surgery rates on the margins. Empirically, we have also already shown that lower liability states do in fact have higher surgery rates [51]. Second, laws on the books for many years are unlikely to affect treatment outcomes such as death or complications except through their effect on the choice of treatment in specific clinical areas.

However, we present both instrumental variable estimation (IV) and ordinary least squares (OLS) reduced form results. Our primary analytical approach is the IV, because it provides estimates generalizable only to the subset of patients in the entire population whose surgery choices are sensitive to the instrument specified [49, 53, 55, 57, 75–83]. Consistent with our research objective, the IV approach explicitly estimates the health outcome impact among the subset of patients whose surgery choice was sensitive to the medical malpractice liability environment. However, for comparison, we also present results from OLS reduced-form estimates obtained by regressing the various health outcomes on the tort rule instruments directly. These results represent the average treatment effect on the treated – that is, the health outcome differences among all shoulder patients across states with different malpractice liability rules.

In this study, our instrumental variables consist of a series of indicator variables for JSLR, contributory negligence, cap on punitive damages, and punitive evidence rule as described above. For our reduced form analyses, we conducted a series of ordinary least squares regressions by regressing our outcomes of interest on the number of liability-reducing tort reform rules and all of the aforementioned exogenous covariates.

We conducted our instrumental variable and reduced form analysis using respectively the procedures IVREG and LM in R statistical software, version 1.4.1106 (Vienna, Austria), controlling for patient demographic and clinical characteristics.

### Statistical analysis

We provide the summary statistics of patient demographic and clinical characteristics for all patients and for patients disaggregated by surgical choice (surgery or conservative management) as well as by residence in states with 0 to 4 tort reform rules. For patients disaggregated by surgical choice, differences in the characteristics were tested using the 2-sample independent t test for continuous variables and Pearson’s χ2 test for categorical data. We used the Cochrane-Armitage test to evaluate trends over patients grouped by the number of provider liability-reducing tort reform rules in the state where the patient resides [84, 85]. For the instrumental variable and reduced form analyses, we ran separate specifications for the overall sample, as well as the various subgroups based on age, CCI, and FRI. We used *P* < 0.05 as the threshold for statistical significance. F statistics from the first-stage regression were used to test the strength of our instruments, or their ability to account for a significant amount of variation in surgery choice in the overall sample population and subgroups. Following standard practices in the literature, we considered an F statistic greater than 10 to be indicative of a strong instrument [86].

This study follows the Strengthening the Reporting of Observational Studies in Epidemiology (STROBE) reporting guideline and was exempted by the University of South Carolina Institutional Review Board as not involving human subjects.

## Results

### Sample characteristics

The final analytical cohort included 67,966 patients, with 50% of the patients aged 80 and above. Only 19% of the patients were male, and the great majority of patients were White (93%). Only about a quarter of the patients had the highest indicators of vulnerability (26% had CCI of 4 plus, and 23% had FRI of 3 plus). Overall, 10% of the subjects died, 52% had a surgery-related complication, and 56% enjoyed treatment success. In the overall sample, patients were subject to an average of 2.64 liability-reducing tort reform rules.

Eighty-four percent of patients received conservative management in the 60 days after index diagnosis, and 16% received early surgery. Descriptive statistics reveal that the conservative management and surgical patients differ in many characteristics, with surgical patients likely to be younger and less clinically vulnerable. The surgical group had a higher proportion of patients with 0 CCI Score (29% versus 25%) and a lower proportion of patients with 4 + CCI (22% versus 27%). Likewise, the surgical group had higher proportions of patients with 0 FRI (42% versus 34%), and a lower proportion of those with high (3 +) FRI (17% versus 24%). These results suggest that the surgery variable could potentially be endogenous.

Table 2 also shows the differences in average unadjusted one-year outcomes by treatment group. The one-year mortality rate for the surgically managed patients was 7%, versus 11% for the conservatively managed patients. However, 59% of the surgical group had a complication in the outcome period, versus 51% among the patients who did not have surgery. And the surgical group had a 43% treatment success rate, versus 59% for the conservative management group. The summary statistics also reveal that patients who ultimately received surgery lived in states with more liability-reducing tort rules than conservatively managed patients, by 2.73 to 2.62.

**Table 2:** Summary Statistics

	Total (*N* = 67,966)	Conservative Management (*N* = 56,880)	Surgery (*N* = 11,086)	*p* value
Tort-related variables				
Number of tort rules	2.64	2.62	2.73	< 0.001
Not comparative negligence	0.52	0.52	0.53	0.817
JSL	0.76	0.76	0.78	< 0.001
Cap on punitive damages	0.66	0.65	0.69	< 0.001
Punitive evidence rule	0.7	0.69	0.73	< 0.001
Geographic variables				
South	0.41	0.41	0.45	< 0.001
West	0.16	0.16	0.17	0.006
Northeast	0.17	0.18	0.12	< 0.001
Mountain West	0.25	0.25	0.26	0.03
Demographic variables				
Male	0.19	0.2	0.18	< 0.001
Age (66–69)	0.14	0.13	0.17	< 0.001
Age (70–75)	0.21	0.2	0.26	< 0.001
Age (76–79)	0.15	0.15	0.18	< 0.001
Age (80–85)	0.25	0.25	0.24	0.001
Age (86 plus)	0.25	0.27	0.16	< 0.001
Asian	0.01	0.01	0.01	0.016
Black	0.03	0.03	0.02	< 0.001
Hispanic	0.01	0.02	0.01	0.001
Other race	0.01	0.01	0.01	0.207
White	0.93	0.93	0.95	< 0.001
Fully dual eligible	0.14	0.14	0.09	< 0.001
Mean spending (county)	9,505.65	9,503.39	9,517.26	0.367
Mean life expectancy (county)	78.35	78.37	78.22	< 0.001
CCI (0)	0.25	0.25	0.29	< 0.001
CCI (1)	0.21	0.2	0.22	0.003
CCI (2)	0.16	0.16	0.15	0.039
CCI (3)	0.12	0.12	0.12	0.49
CCI (4 plus)	0.26	0.27	0.22	< 0.001
FRI (0)	0.35	0.34	0.42	< 0.001
FRI (1)	0.26	0.26	0.27	0.032
FRI (2)	0.16	0.16	0.14	< 0.001
FRI (3 plus)	0.23	0.24	0.17	< 0.001
Clinical variables (prev. history)				
Osteoarthritis	0.25	0.25	0.23	< 0.001
Rheumatoid arthritis	0.08	0.08	0.07	0.025
Rotator cuff arthropathy	0.07	0.07	0.06	0.199
Avascular necrosis	0	0	0	0.013
Outcome variables				
Death (365 days)	0.1	0.11	0.07	< 0.001
Any complications (365 days)	0.52	0.51	0.59	< 0.001
Treatment Success	0.56	0.59	0.43	< 0.001

Table 3 compares patient characteristics across states with different number of tort reform rules, which served as our instrumental variables. The overall surgery rate is 16%, and the data show that surgery rates increased monotonically with an increasing number of tort reform rules – from 9 to 14%, 15%, 17%, and 18% for patients living states with 0, 1, 2, 3, and 4 tort reform rules that decrease medical malpractice liability pressure. This increase is statistically significant at *p* < 0.001 according to the Cochran-Armitage test of trends. Unadjusted mortality rates increase slightly with more malpractice liability-reducing tort reform rules, from 9% for patients in states with 0 or 1 such rule, to 10% or 11% in states with 2 or more rules (*p* < 0.01). Unadjusted measured patient characteristics associated with study outcomes, such as CCI score and FRI score were similar across number of state tort reform rules, except for the highest CCI and FRI scores. For differences that achieved statistical significance, most were small compared to the differences between the surgically and conservatively managed patients in Table 2.

**Table 3:** Summary Statistics by Number of Liability-Reducing Tort Reform Rules

		Number of Tort Rules that Reduce Medical Malpractice Liability					
	Total (*N* = 67,966)	0 Tort Rule (*N* = 1999)	1 Tort Rule (*N* = 3135)	2 Tort Rules (*N* = 20,200)	3 Tort Rules (*N* = 34,629)	4 Tort Rules (*N* = 8003)	*p* value
Intervention variable							
Surgery	0.16	0.09	0.14	0.15	0.17	0.18	< 0.001
Geographic variables							
South	0.41	0	0.29	0.33	0.41	0.8	< 0.001
West	0.16	0	0.05	0.03	0.3	0	< 0.001
Northeast	0.17	1	0.66	0.24	0.08	0	1
Mountain West	0.25	0	0	0.4	0.21	0.2	1
Demographic variables							
Male	0.19	0.2	0.18	0.19	0.19	0.2	0.36
Age (66–69)	0.14	0.11	0.12	0.13	0.14	0.13	< 0.001
Age (70–75)	0.21	0.19	0.21	0.2	0.22	0.21	< 0.001
Age (76–79)	0.15	0.15	0.14	0.15	0.15	0.15	0.43
Age (80–85)	0.25	0.27	0.25	0.25	0.24	0.25	1
Age (86 plus)	0.25	0.27	0.28	0.26	0.25	0.26	1
Asian	0.01	0.01	0	0.01	0.01	0	0.002
Black	0.03	0.01	0.01	0.04	0.03	0.03	< 0.001
Hispanic	0.01	0.01	0	0.01	0.02	0.02	< 0.001
Other race	0.01	0.01	0	0.01	0.01	0.01	0.061
White	0.93	0.97	0.98	0.94	0.92	0.94	1
Fully dual eligible	0.14	0.15	0.13	0.12	0.15	0.12	0.46
Spending (1st quintile)	0.21	0.01	0.21	0.25	0.23	0.03	1
Spending (2nd quintile)	0.21	0.2	0.39	0.26	0.19	0.05	1
Spending (3rd quintile)	0.19	0.38	0.31	0.19	0.17	0.22	1
Spending (4th quintile)	0.2	0.4	0.07	0.22	0.17	0.24	1
Spending (5th quintile)	0.2	0	0.02	0.08	0.24	0.47	< 0.001
Life expectancy (1st quintile)	0.2	0	0.14	0.17	0.22	0.23	< 0.001
Life expectancy (2nd quintile)	0.19	0	0.15	0.2	0.21	0.21	< 0.001
Life expectancy (3rd quintile)	0.2	0.07	0.21	0.22	0.2	0.18	0.039
Life expectancy (4th quintile)	0.19	0.4	0.34	0.21	0.17	0.15	1
Life expectancy (5th quintile)	0.21	0.53	0.16	0.2	0.2	0.22	1
CCI (0)	0.25	0.27	0.26	0.26	0.26	0.23	1
CCI (1)	0.21	0.21	0.21	0.21	0.21	0.2	0.98
CCI (2)	0.16	0.15	0.16	0.16	0.15	0.17	0.062
CCI (3)	0.12	0.12	0.13	0.12	0.12	0.13	0.24
CCI (4 plus)	0.26	0.25	0.24	0.25	0.26	0.27	< 0.001
FRI (0)	0.35	0.34	0.34	0.36	0.35	0.33	0.96
FRI (1)	0.26	0.27	0.26	0.27	0.26	0.26	0.96
FRI (2)	0.16	0.17	0.17	0.16	0.15	0.16	0.78
FRI (3 plus)	0.23	0.22	0.23	0.21	0.23	0.25	< 0.001
Clinical variables							
Osteoarthritis	0.25	0.22	0.21	0.24	0.25	0.28	< 0.001
Rheumatoid arthritis	0.08	0.08	0.08	0.08	0.08	0.09	0.35
Rotator cuff arthropathy	0.07	0.06	0.07	0.06	0.07	0.06	0.041
Avascular necrosis	0	0	0	0	0	0	0.32
Outcome variables							
Death (365 days)	0.1	0.09	0.09	0.1	0.11	0.1	0.006
Any complications (365 days)	0.52	0.55	0.52	0.51	0.52	0.54	0.057
Treatment Success	0.56	0.53	0.57	0.56	0.56	0.56	0.082

### Instrumental variable results

Table 4 Panel A presents the estimates from our instrumental variable regressions. The first column contains the F statistics from our first-stage regressions. In the instrumental variable literature, instruments with an F statistic less than 10 from the first-stage regression are considered weak and more susceptible to bias [87]. Except the subgroup of patients with CCI of less than 2, all of our F statistics are greater than 10. (Total cohort, 34.71; age < 80 years, 13.07; age >  = 80 years, 25.73; CCI score >  = 2, 28.4; FRI score < 2, 22.13; FRI score >  = 2, 13.52), signifying that our tort reform rules were sufficiently strong instruments in almost all patient groups. In the third column, Table 4 provides the mean percentage of patients who underwent surgery in each subgroup and the respective range in rates across the states with different numbers of tort reform rules. The variation in surgery rates is the range within which our instrumental variable estimates apply. Moreover, our subgroups show that the surgery rates this column were lower for vulnerable groups. Surgery rates were 20% for those < 80 years but 13% for those >  = 80 years; 18% for those with CCI < 2 and 15% for those with CCI >  = 2; and 18% for those with FRI < 2 and 13% for those with FRI >  = 2.

**Table 4:** Estimates of the Association of Surgery with Adverse Events and Mortality for Medicare Patients With Proximal Humeral Fracture in 2011 and Stratified Subgroups

Estimate	F Statistic	Surgery Rate Across Tort Law Regions, Mean (Range), %^a^	Success Rate, %	Adverse Event Rate, %	Mortality Rate, %
Panel A: Instrumental Variable Estimates					
Total Cohort (*N* = 67,966)	34.71***	0.16 (0.09—0.18)	0.32*	-0.1	0.21**
Age < 80 (*N* = 33,896)	13.07***	0.2 (0.14—0.22)	0.89***	-0.14	0.28**
Age > = 80 (*N* = 34,070)	25.73***	0.13 (0.05—0.15)	-0.01	-0.05	0.13
Charlson Comorbidity Index Score					
< 2 (*N* = 31,270)	9.96***	0.18 (0.1—0.19)	0.31	-0.23	0.06
> = 2 (*N* = 36,696)	28.4***	0.15 (0.08—0.17)	0.19	0.05	0.29**
Frailty Index Score					
< 2 (*N* = 41,860)	22.13***	0.18 (0.1—0.2)	0.54***	-0.15	0.08
> = 2 (*N* = 26,106)	13.52***	0.13 (0.07—0.15)	-0.24	0	0.45**
Panel B: Reduced-Form Estimates^b^					
Total Cohort (*N* = 67,966)			0.002	-0.001	0.004***
Age < 80 (*N* = 33,896)			0.004	-0.002	0.005***
Age > = 80 (*N* = 34,070)			0.0001	-0.001	0.003
Charlson Comorbidity Index Score					
< 2 (*N* = 31,270)			0.01*	-0.004	0.001
> = 2 (*N* = 36,696)			-0.002	0.001	0.01***
Frailty Index Score					
< 2 (*N* = 41,860)			0.01**	-0.003	0.001
> = 2 (*N* = 26,106)			-0.01*	0.0004	0.01***

^a^Surgery rate across states grouped from the lowest to highest number of liability-reducing tort laws

^b^Reduced form estimates the policy effect for each additional liability-reducing tort law rule

^***^ *p* < 0.001, ** *p* < 0.001, * *p* < 0.05

Instrumental variable estimates reveal that in the overall sample, there was a tradeoff between mortality and treatment success in the marginal patients whose surgery choices were sensitive to malpractice liability-reducing tort rule environment. Patients in states with lower provider malpractice liability risk were more likely to have both higher treatment success rates and higher mortality. In the full cohort, the instrumental variable results show a 1–percentage point increase in surgery rates was associated with a 0.32–percentage point increase in the 1-year treatment success rates (*P* < 0.05) *and* a 0.21-percentage point increase in the 1-year mortality rates (*P* < 0.001).

When we disaggregated our analyses into subsets of patients with different clinical characteristics, we found that there was a split in tradeoffs between detriments and success based on what is likely to be salient (age) versus less salient (CCI, FRI) vulnerability status. For the subgroups based on age, detriments and success were concentrated in the younger group (< 80 years), which experienced both an increase in treatment success (0.89 percentage point, *p* < 0.001) and mortality (0.28 percentage point, *p* < 0.001). For the subgroups based on less salient vulnerability status, the less vulnerable group had an increase in treatment success, respectively by 0.54 percentage-point (*p* < 0.001) and 0.31 percentage-point increase (not statistically significant) for those with FRI < 2 and CCI < 2. However, the more vulnerable groups experienced the detriments – among patients with CCI >  = 2 and FRI >  = 2, mortality rates increased respectively by 0.29 percentage point (*p* < 0.01) and 0.45 percentage point (*p* < 0.01).

### Reduced form results

Our reduced form results are largely consistent with the results from our IV regressions. See Panel B “Reduced Form Estimates” in Table 4. In the analyses performed on the entire group of shoulder patients, the states with more malpractice liability-reducing rules had higher mortality rates among all shoulder patients: Each additional rule is associated with a 0.004 point increase in mortality (*p* < 0.001). Our subgroup analyses reveal similar patterns as the IV results, that more vulnerable patients (CCI >  = 2 and FRI >  = 2) generally had higher mortality in states with more liability-reducing rules (both 0.001, *p* < 0.001). As in the IV results, in the subgroup analyses by age, the older group (> = 80) did not experience statistically significant mortality increase in the states with laxer malpractice environment, but the younger of the elderly (< 80) did (0.005, *p* < 0.001). In terms of treatment success, the reduced form results also showed that less vulnerable patients enjoyed the benefit from surgery (for CCI < 2, an increase of 0.01 in treatment success (*p* < 0.05), and for FRI < 2, an increase of 0.01, (*p* < 0.01)), but not the more vulnerable patients.

We also conducted a separate series of regression (unreported) using each malpractice liability-reducing rule individually as independent variables in our reduced form regressions, and found that cap on punitive damages and the punitive evidence rule had larger coefficient estimates than JSL and contributory negligence.

We note that the reduced form point estimates are much smaller than the IV estimates, not surprising because our reduced form results represent the average over all shoulder patients for each additional liability-reducing rule, who may or may not have undergone surgery or received any treatment at all. On the other hand, the IV results apply only to a smaller set of marginal patients, representing the outcomes associated with each additional percentage of surgery among patients whose surgery choice was sensitive to the medical malpractice liability environment.

## Discussion

Our study results reveal two overarching patterns. First, increased surgery rates associated with malpractice liability-reducing tort rules yielded a tradeoff in terms of positive and negative health outcomes. Second, the stratified subgroup analyses in the instrumental variable regressions revealed that the tradeoff depended on the saliency of the vulnerability, with different patient subgroups enjoying the benefit and experiencing the detriments from surgery differentially. Our reduced form results are largely consistent with our IV results.

Using tort reform rules as instrument variables, we focused on the effect of surgery among those patients whose surgery was sensitive to differential surgery rates associated with the state tort law environment. These results are likely the most policy relevant in the context of our research question. Surgeons are not likely to begin performing surgery indiscriminately even when their liability for negligence is reduced. Therefore, understanding the health outcome impact on the set of patients whose surgery was potentially influenced by the relaxation of tort liability rules is a more appropriate measure of the potential effect of tort law reform. Our previous work has shown that liability-reducing rules are on average associated with a 1-to 2-percentage point increase in surgery rates [51], and our instrumental variable results found that surgery associated with tort reform laws increased treatment success rates by 0.32-percentage point overall, but also increased mortality rates by 0.21-percentage point for every 1 percentage-point increase in surgery rates.

What, then, is the type of patients who are on the extensive margin? Our subgroup analyses of unadjusted surgery rates across states with differing number tort rules reveal that surgeons did in fact choose healthier patients – surgery rates were higher among those who were < 80 versus >  = 80, CCI < 2 versus >  = 2, and FRI < 2 versus >  = 2. In a survey of physicians, Studdert et al. described patients that physicians avoided in areas where malpractice liability risks are high: Patients who had complex medical conditions, or who were perceived to be litigious [88]. The physicians surveyed also expressed that they eliminated procedures that were prone to complications such as surgery. It is possible, then, that the surgeons who face lower malpractice liability pressure dipped into the margins of such patients, with a tradeoff in outcomes between detriments and benefits. In fact, we performed a naïve OLS regression of health outcomes on the endogenous variable “surgery,” (results not reported) our estimated signs flip from the IV regression – surgical patients had lower mortality in this naïve regression, suggesting overall, that surgeons chose healthier patients and that surgery choice was likely correlated to unobservable factors in the error term that were in turn also correlated with health outcomes.

Our subgroup analyses further revealed that patients with different levels of saliency in vulnerability or risk bore the tradeoffs unequally. More vulnerable patients in states with more malpractice liability-reducing rules had higher mortality effects associated with surgery (CCI >  = 2 and FRI >  = 2), and less vulnerable patients (FRI < 2) had higher treatment success rates. However, the exception to this pattern occurred among younger Medicare patients (< 80 years), who both benefited from higher treatment success rates and experienced higher mortality rates. It is possible that age is a far more salient vulnerability indicator than comorbidity or function status, so surgeons were careful to avoid selecting older patients who may be particularly vulnerable to the risk of death. It is also likely that among those aged < 80 years, the risks and benefits may have accrued to even finer subgroups (such as < 80 years and FRI >  = 2 versus < 80 years and FRI < 2). Future research should consider the surgical outcomes of patients with different combinations of clinical and demographic characteristics, whose surgery was sensitive to tort reform rules.

Our results contrast with many studies that find little to no effect of tort liability rules on health outcomes [7, 26, 27, 89]. However, many of these studies focus on child birth[26, 27] or non-surgical chronic medical conditions [7, 89]. Our study population, consisting of elderly Medicare beneficiaries who suffered a bone fracture, may be particularly vulnerable to the adverse effects of surgery. In this context, external non-clinical factors that influence treatment have a greater probability of perturbing the balance in the treat-no treat decision. A comparison of the study results from different medical areas attests to the importance of understanding the medical liability environment in diverse clinical contexts. There is likely no one-size-fits-all approach to medical malpractice liability reform. Physicians in different areas of practice may make different treatment decisions when balancing risks and benefits and may not make these decisions similarly, especially when there is a lack of level-I clinical guidance as in orthopaedics.

Our study shows that there may be a tradeoff in lower medical malpractice liability for physicians. Beyond this finding, however, it is important to note that if the goal is to reduce the economic and noneconomic toll of the medical malpractice system, shared decision-making may be able to reduce patients’ perception of fault and liability in the setting of an adverse outcome. Better communication and shared decision-making may improve the physician–patient relationship and medical care in general, beyond the immediate goal of reducing medical malpractice litigation [90–92].

Finally, our study also reveals important differences in interpreting results from different methodological approaches. It is essential to conceptualize the theoretical framework in which an intervention is likely to affect outcomes. In this work, we made three plausible assumptions about how liability rules may affect health outcomes in a clinical specialty in which surgery is one of two major choices of treatment, the other being conservative management. First, we believe that most of the benefits and harms in this group of patients will be determined by how likely patients undergo surgery because liability pressure is likely to affect outcomes through surgery choice. Second, we believe that there is treatment effect heterogeneity, i.e., patients receiving identical treatment may have differential health outcomes. Third, essential heterogeneity exists, such that surgeons will not randomly choose additional patients to recommend surgery in a lower liability environment and will specifically select patients who are more likely, based on experience, to benefit from the surgery.

In other words, only a subset of all shoulder patients would be influenced by the liability regime in each state. For these reasons, the IV results most closely resemble how differences in the liability environment may affect health outcomes. Anecdotally, we were also informed by our clinician co-investigators that liability rules do affect their treatment decisions in marginal cases. On the other hand, our reduced form results represent the average treatment effect on the treated, capturing differences in health outcomes among all shoulder patients between states with different liability environments, including patients whose surgery choice would not have been influenced by the medical malpractice liability environment. Nevertheless, both our IV and reduced form results demonstrate similar associations between medical malpractice liability and patient health outcomes.

### Limitations

In interpreting our results, the following caveats should be noted. First, our work properly identifies the treatment outcomes due to surgery choice sensitive to differential surgery rates associated with the state tort law environment in 2011. However, we are unable to conclude that the differences in surgery rates across higher and lower medical malpractice liability environments were entirely caused by differences in the malpractice environment. We are also not able to state that switching liability rules today will change practice patterns in the short or even medium term. Indeed, it is possible that differences in surgery rates that we observe in 2011 simply reflect practice patterns that predate the medical liability reforms in the 1990s. On the other hand, there are theoretical reasons to believe that medical malpractice rule changes have the potential to change how physicians choose to recommend surgery in the longer term. Lower liability suggests lower risk for surgeons from negligent harm caused to the patient. Over time, existing physicians may be willing to undertake greater risk if the consequences of such risk are less severe. New physicians who have more aggressive practice styles may be attracted to the lower liability environment.

Without empirical data from 20 years ago, it is not feasible to make conclusions on causality. However, our belief is that the truth likely lies somewhere between the two extreme possibilities (that differences in surgery rates are due entirely to differences in malpractice rules, or none of the differences can be attributed to the malpractice liability rules). As a result, we believe that our results can fairly be considered an upper bound of the impact of the medical malpractice liability environment on the surgical outcomes among patients whose surgery choice was sensitive to the legal environment.

Related to the causality concern raised above, we were not able to account for all factors that are associated with surgery and the treatment choice, such as fracture complexity. Of particular concern for our study design is the possibility that these unmeasured factors are distributed differently across patients in states with different tort law environments. For the variables that we do observe, however, the broad geographic distribution of states with different number of tort reform rules in Fig. 1, and the lack of statistical significance in the differences across states with different tort environments in most measured patient characteristics (except in the extreme values) in Table 3, suggest that patients were fairly similar across states. For the variables that do differ between states, the Cochran-Armitage tests suggest that states with more liability-reducing tort rules have patients who have higher baseline medical expenditures, low baseline life expectancies, higher Charlson Comorbidity Index and higher Function-Related Indicator scores. In other words, despite having patients who are less ideal surgical candidates, states with more liability-reducing tort reform rules actually have higher surgery rates, a pattern that is consistent with the laws having a strong effect on surgery rates. We also directly controlled for these observable differences in our regressions.

In addition, the estimates on treatment success should be interpreted with caution. It is possible that surgeons acted with greater caution when treating conservative management patients (in both the initial treatment window and subsequent outcome window), making their outcomes appear better than they actually were. This result is possible because our measure for treatment success depends on providing services that may have been withheld among these patients. If so, this scenario may bias our results with respect to increased treatment success among patients whose surgery choice was sensitive to differential medical malpractice environments.

Also, estimates on our outcomes should be interpreted only within the surgery rate ranges described in Table 4. However, for the purposes of our study, these are likely the ranges most relevant to our study objective, as they represent the surgery rate ranges sensitive to different tort law environments. Moreover, our study sample dated from 2011, and surgery rates for PHF the relevant ranges have likely changed in the interim. Estimates based on newer data will likely differ based on these changes.

We also excluded patients who did not survive past the treatment period. Therefore, our results do not generalize to those very frail patients. Finally, these results should be interpreted with caution in the broader international context. It is commonly acknowledged that the practice of medicine is subject to far more litigation in the United States than the rest of the world [93–101]. It is therefore possible that the specific effects that we found in this study may be higher in magnitude or may exist only in the United States. However, there is also a large body of research studying the impact of malpractice liability on physician treatment choices in other parts of the world [See, e.g., 95–101] It may therefore be of interest to replicate this study in non-American health systems to ascertain whether medical malpractice liability exerts a heterogeneous effect on patient populations elsewhere as well.

## Conclusion

Using a national sample of patients with PHF, we investigated the health outcome results of surgery across states with different medical malpractice liability environments. We found a 0.32 percentage-point increase in treatment success rates and a 0.21 percentage-point increase in mortality rates for every 1-percentage point increase in surgery among patients in states with lower versus higher malpractice liability pressure. When we disaggregated patients into subgroups, health outcomes split between salient (age) versus less salient vulnerabilities (CCI, FRI). Detriments and benefits accrued only to patients under 80 in the age-based subgroups. However, for the subgroups with the less salient vulnerabilities, mortality increased among the more vulnerable and treatment success increased among the less vulnerable. These results suggest that in states where medical malpractice practice liability was lower, orthopaedic surgeons may have expanded their set of surgery patients to those at higher risk for death, particularly for those whose risks are less saliant. Overall, differential medical malpractice liability risks across states were associated with contradictory consequences for health. Future studies should analyze whether observed differences in surgery rates between states are causally related to differences in the liability environments to conclude that changing liability rules could result in the health outcome consequences that we observed. In addition, because our study results could change based on evolving advancing in medical care and different clinical and legal contexts, future analysis should also be conducted to investigate the health outcomes associated with the medical liability environment using updated data, as well as in other medical specialties.

## Supplementary Information

**Additional file 1: Supplementary Appendix Table 1. **Inclusion and Exclusion Criteria. **Supplementary Appendix Table 2. **Full Description of Variables.

## Data Availability

The Medicare claims data underlying our study can be obtained from ResDAC upon successful application, at https://www.resdac.org. The Database of State Tort Law Reforms, 1980–2018 (6^th^ Edition) from the University of Texas School of Law can be accessed at https://law.utexas.edu/faculty/ravraham/dstlr.php.

## Abbreviations

PHF: Proximal Humeral Fracture
ResDAC: Research Data Assistance Center
JSLR: Joint and Several Liability Reform
STROBE: Strengthening the Reporting of Observational Studies in Epidemiology
CCI: Charlson Comorbidity Index
FRI: Function-Related Indicators
