# Supplementary material for: What are the health consequences associated with differences in medical malpractice liability laws? An instrumental variable analysis of surgery effects on health outcomes for proximal humeral facture across states with different liability rules
Source: BMC Health Serv Res. 2022 May 3;22:590. doi: 10.1186/s12913-022-07839-0 (PMC9063084; doi:10.1186/s12913-022-07839-0)
Supplement: Supplementary file 1 — Additional file 1: Supplementary Appendix Table 1. Inclusion and Exclusion Criteria. Supplementary Appendix Table 2. Full Description of Variables. [file 12913_2022_7839_MOESM1_ESM.docx]

**What are the Health Outcome Consequences of Reduced Medical Malpractice Liability?**

**An Instrumental Variable Analysis of Differences in Health Outcomes following Treatment for Proximal Humeral Facture in States that Adopted Different Numbers of Tort Reform Rules**

**Brian Chen***

915 Greene Street Suite 354, Columbia, SC 29205 USA

Department of Health Services Policy and Management, University of South Carolina

**Sarah Floyd**

116 Edwards Hall, Clemson, SC 29634

College of Behavioral, Social and Health Sciences, Clemson University

**Dakshu Jindal**

915 Greene Street, Columbia, SC 29205 USA

Department of Health Services Policy and Management, University of South Carolina

**Cole Chapman**

345 CPB, 180 South Grand Ave, Iowa City, Iowa 52242 USA

Department of Pharmacy Practice and Science, University of Iowa

**John Brooks**

915 Greene Street Suite 302, Columbia, SC 29205 USA

Department of Health Services Policy and Management, University of South Carolina

*Corresponding Author. Please send all correspondences to [bchen@mailbox.sc.edu](mailto:bchen@mailbox.sc.edu)

**Appendices**

**Supplementary Appendix Table 1: Inclusion and Exclusion Criteria**

*Medicare 2011 PHF Sample Inclusion Criteria*

| **Inclusion Criteria** | **N** |
| --- | --- |
| Medicare Part B carrier (physician services), outpatient, or MEDPAR (inpatient) claims with a proximal humerus fracture diagnosis from January 1, 2011-December 31, 2011 (ICD-9 Diagnosis codes: 812.00, 812.01, 812.02, 812.09, 812.10, 812.11, 812.12, 812.13, 812.19) (Index diagnosis) | 130,959 |
| No Part B carrier, outpatient, or MEDPAR claims with proximal humerus fracture diagnosis in 365-days before the index diagnosis in 2011 | 107,838 |
| Shoulder x-ray claim (HCPCS codes: 73000, 73010, 73020, 73030, 73050, 73060) in Part B carrier or outpatient revenue center claims within 7 days of index diagnosis (x-ray claim can occur before or after index diagnosis) | 95,229 |
| No Part B carrier, outpatient, or MEDPAR claims with a diagnosis of clavicle or hip fracture within 7 days of index diagnosis | 86,147 |
| No Part B carrier, outpatient, or MEDPAR claims with total joint replacement procedure in 365-days before the index diagnosis in 2011 | 85,841 |
| Age 66+ at index diagnosis | 84,589 |
| Located within continental United States or Hawaii | 84,399 |
| Continuously enrolled in Medicare Parts A and B and never enrolled in HMO, from 365-days prior to index to 365-days after index diagnosis | 77,075 |
| Complete HRR data | 77,053 |
| Complete PHF data after merging with State Tort Rules | 67,966 |

**Supplementary Appendix Table 2: Full Description of Variables**

| **Variable/Concept** | **Time Frame** | **Source** | **Qualifying Codes** | **Data Files** |
| --- | --- | --- | --- | --- |
| Emergency department and ambulance use near index shoulder complaint | Index date and day prior | Revenue Center codes and Berenson-Eggers Type of Service Codes | (Revenue Center Codes: 0450, 0451, 0452, 0456, 0459, 0981, 0540, 0541, 0542, 0543, 0544, 0545, 0546, 0547, 0548, or 0549) and (Berenson-Eggers Type of Service Codes beginning with: M3 or A01) | Outpatient revenue center, Carrier |
| MRI of the upper extremity or orbit, face and neck | Within 90 days of index date | HCPCS codes | **70540, 70542, 70543, 73218, 73219, 73220, 73221, 73222, or 73223** | Carrier (Part B Physician Claims), Outpatient revenue center |
| Diagnosis of PHF | Within 7-days of the earliest dated X-ray | ICD-9 Diagnosis Code | **812.00, 812.01, 812.02, 812.09, 812.10, 812.11, 812.12, 812.13, 812.19 (any DX on the claim)** | Carrier (Part B Physician Claims), **Outpatient base claims** |
| Surgical PHF Repair | Surgery claim within the 60-day treatment exposure period after the MRI and diagnosis of an ARCT and no earlier or concurrent claim for PT | ICD-9 Procedure codes or HCPCS codes | **HCPCS codes (23630, 23615, 23670, 23680, 23472, 23470, 23616)** |  |
| Patient Age | On index date | Date of birth filed with Medicare |  | Medicare Beneficiary A/B Summary File |
| Frailty Index (FRI) | All claims over period one year prior to index date | Chrischilles EA, Schneider KM, Wilwert J, Lessman G, O’Donnell B, et al. “Beyond comorbidity: Expanding the definition and measurement of complexity among older adults using administrative claims data.” *Medical Care. 2014;* 52(3), S75-84.  Chrischilles EA, Schneider KM, Shroeder M, Letuchy E, Wallace RB, Robinson JG. “Association of pre-admission functional status with use and effectiveness of secondary prevention medications in elderly survivors of acute myocardial infarction.” *J of the American Geriatrics Society*, 2016; 64(3): 526-535. DOI: 10.1111/jgs.13953 | | |
| Charlson Comorbidity Index (CCI) | All claims over period one year prior to index date | Charlson ME, Pompei P, Ales KL, et al. A new method of classifying prognostic comorbidity in longitudinal studies: development and validation. Journal of chronic diseases 1987;40:373-383 | | |
| Sum Total Payments made by Medicare and Beneficiary to Providers in 365 days prior to index | All claims over period one year prior to index date | Line Payment Amounts | N/A | Inpatient, Outpatient, Durable Medical Equipment, Home Health, Carrier (Physician services), skilled nursing |
| Gender | 2011 | Gender filed with Medicare in 2011 | N/A | Medicare Beneficiary A/B Summary File |
| Race | 2011 | Race filed with Medicare in 2011 | N/A | Medicare Beneficiary A/B Summary File |
| Medicaid Dual Eligible Status | Index Month | Dual-eligibility status in month of index (not dual eligible, partial dual-eligible, or fully dual-eligible) | Dual-Status code of 01 through 06, or 08. | Medicare Beneficiary Part D Components File |
